# Calcium/calmodulin-mediated microbial symbiotic interactions in plants

**DOI:** 10.3389/fpls.2022.984909

**Published:** 2022-10-18

**Authors:** Peiguo Yuan, Feixiong Luo, Cynthia Gleason, B. W. Poovaiah

**Affiliations:** ^1^ Department of Plant Pathology and Microbiology, Texas A&M University, College Station, TX, United States; ^2^ Department of Pomology, Hunan Agricultural University, Changsha, China; ^3^ Department of Plant Pathology, Washington State University, Pullman, WA, United States; ^4^ Department of Horticulture, Washington State University, Pullman, WA, United States

**Keywords:** Ca^2+^ signaling, local and systematic signaling, mycorrhizal development, plant-beneficial microbe interaction, rhizobial nodulation

## Abstract

Cytoplasmic calcium (Ca^2+^) transients and nuclear Ca^2+^ oscillations act as hubs during root nodulation and arbuscular mycorrhizal symbioses. Plants perceive bacterial Nod factors or fungal signals to induce the Ca^2+^ oscillation in the nucleus of root hair cells, and subsequently activate calmodulin (CaM) and Ca^2+^/CaM-dependent protein kinase (CCaMK). Ca^2+^ and CaM-bound CCaMK phosphorylate transcription factors then initiate down-stream signaling events. In addition, distinct Ca^2+^ signatures are activated at different symbiotic stages: microbial colonization and infection; nodule formation; and mycorrhizal development. Ca^2+^ acts as a key signal that regulates a complex interplay of downstream responses in many biological processes. This short review focuses on advances in Ca^2+^ signaling-regulated symbiotic events. It is meant to be an introduction to readers in and outside the field of bacterial and fungal symbioses. We summarize the molecular mechanisms underlying Ca^2+^/CaM-mediated signaling in fine-tuning both local and systemic symbiotic events.

## Introduction

Sessile plants have evolved complex signaling networks to cope with various environmental changes (Laplaze et al., 2015; Tian et al., 2020). Calcium (Ca^2+^) signals play a central role in the networks that regulate various physiological responses of all eukaryotes, including plants (Berridge et al., 2003; Yuan et al., 2018a; Yuan et al., 2018b; Luan and Wang, 2021). Ca^2+^ signaling is also crucial in plant-pathogen interactions. Ca^2+^ influxes are induced when plants perceive pathogen-/microbe-associated molecular patterns (PAMPs/MAMPs) through cell surface pattern recognition receptors (PRRs) to trigger basal defense responses. For example, the plant plasma membrane receptor flagellin-sensitive 2 (FLS2) recognizes the conserved bacterial PAMP, flg22, to induce transient Ca^2+^ influxes (Aslam et al., 2008; Ma et al., 2017; Yuan et al., 2020). A stronger and prolonged Ca^2+^ signature occurs when the bacterial pathogen *Pst* DC3000 carrying the effector *AvrRpt2* is recognized in resistant plants, as compared to *Pst* DC3000 without this avirulent factor (Yuan et al., 2021). In another example of calcium’s role in plant resistance, the resistance protein ZAR1 forms a protein complex that triggers sustained calcium ion influx into the cell that subsequently leads to cell death and immune responses (Bi et al., 2021). The pathogen triggered Ca^2+^ signaling is perceived and relayed by various Ca^2+^ receptors, such as CaMs/calmodulin-like proteins (CMLs), calcineurin B-like protein (CBL)-CBL-interacting protein kinases (CIPK) and Ca^2+^calcium-dependent protein kinases (CDPKs or CPKs) (Tian et al., 2020; Köster et al., 2022; Yuan and Poovaiah, 2022). Interestingly, Ca^2+^ also plays a critical role in the symbiotic relationship between plants and beneficial microbes. The role of Ca^2+^ has been well described in legumes forming a symbiotic relationship with rhizobia bacteria and arbuscular mycorrhizal fungi.

In the *Medicago-*rhizobial symbiotic relationship, the symbiosis signaling pathway is initiated when the plant receptor complex LysM receptor kinase 3 (LYK3)-Nod factor perception (NFP) recognizes lipo-chitooligosaccharide signals (i.e., Nod factors) from rhizobial bacteria (Haney et al., 2011; Kang et al., 2011). The Does not Make Infections 2 (DMI2)/nodulation receptor-like kinase (NORK), also known as Symbiosis receptor kinase (SYMRK) in *L. japonicus*, interacts with the LYK3-NFP receptor complex; the DMI2-LYK3-NFP protein complex regulates rhizobial infection and nodule development (Ané et al., 2004; Lefebvre et al., 2010). DMI2 interacts with 3-hydroxy-3-methylglutaryl CoA reductase 1 (MtHMGR1), which is a key enzyme in the biosynthesis of many isoprenoid compounds, including cytokinin and mevalonate. Mevalonate is a secondary messenger, and the activation of the mevalonate pathway is important for the activation of the common symbiotic pathway (Kevei et al., 2007; Oldroyd, 2013; Venkateshwaran et al., 2015). The recognition of Nod factors (NFs) by the plant cells activates Ca^2+^ channels, such as the cyclic nucleotide gated channel 15 (CNGC15a, b, c) and DMI1 [which was initially reported as a potassium channel (Ané et al., 2004; Peiter et al., 2007)]. Nod factor recognition also activates the Ca^2+^ pump, a membrane Ca^2+^-ATPase 8 (MCA8). As a result, sharp oscillations of cytoplasmic and perinuclear Ca^2+^ occurs, a phenomenon called Ca^2+^ spiking (Ehrhardt et al., 1996; Wang et al., 2022). Following Nod factor induced Ca^2+^ influxes, some Ca^2+^ binding proteins, such as CCaMK, decode the symbiotic Ca^2+^ signal into down-stream phosphorylation events (Gleason et al., 2006). The Ca^2+^ and CaM-binding CCaMK phosphorylates transcription factors and induces symbiotic-related gene expression to initiate nodulation.

## Ca^2+^-mediated local symbiotic signaling

### Ca^2+^ mediates signal exchange between host and microbe

The first step in root symbiosis is the molecular signal exchange between roots and nitrogen-fixing rhizobia or mycorrhizae. Legume-derived flavonoids induce the biosynthesis of Nod factors in rhizobia, and some symbiosis-related flavonoids are accumulated at the colonization site. This suggests that Nod factors promote flavonoid biosynthesis in a positive feedback loop (Liu and Murray, 2016; Panche et al., 2016; Sharma et al., 2020). These root-excreted flavonoids serve as chemo-attractants to facilitate the movement of rhizobia (e.g., *Sinorhizobium meliloti*) to root hairs (Hassan and Mathesius, 2012). Interestingly, some specific host flavonoids, such as luteolin and naringenin, were shown to induce Ca^2+^ transients in rhizobia, which subsequently activates bacterial Nod-related genes (i.e., *nodA, nodB, and nodC*) in *Rhizobium leguminosarum* cv. *viciae* (Moscatiello et al., 2010; Cui et al., 2019). These findings suggest Ca^2+^ signaling in bacterial symbionts plays a role during plant symbiotic microbe interaction. When studying arbuscular mycorrhizal fungi (AMF)-peanut symbiosis, it was noted that exogenous Ca^2+^ application improved the colonization of peanut roots by AMF and induced the expression of plant genes, including those genes regulating flavonoid biosynthesis (Cui et al., 2019). The results from these studies suggest Ca^2+^ signaling is important during the initial interactions between plants and symbionts.

### Plants perceive microbes *via* symbiotic microbe-induced cytoplasmic and nuclear Ca^2+^ transients

Nod factor-induced Ca^2+^ spikes in root hairs is essential for plant root-nodule symbiosis. Earlier studies have revealed that *S. fredii*-derived NGR234 Nod factor was able to induce Ca^2+^ concentration increases within root hairs of nodulating legumes, such as *Chamaecrista fasciculata*, *Acacia retinoides*, *Cytisus proliferus*, *Lupinus pilosus*, and *Medicago truncatula* (Granqvist et al., 2015). However, Nod factor failed to trigger Ca^2+^ oscillations in the non-nodulating legume *Cercis siliquastrum* (Granqvist et al., 2015). This observation suggests that Ca^2+^ transients are common events in rhizobia-compatible plants. To further investigate Ca^2+^ transients in non-leguminous plants, Granqvist et al. (2015) observed that non-leguminous plants (e.g., *Parasponia andersonii*) could form a symbiotic relationship with rhizobia and exhibit Ca^2+^ spiking in response to NGR234 Nod factors. However, *Trema tomentosa*, a non-nodulating plant related to *Parasponia*, did not exhibit Nod factor-induced Ca^2+^ oscillations (Granqvist et al., 2015). These results suggest that Nod-factor-triggered Ca^2+^ oscillations are a common feature in response to symbiotic bacteria in nodulating species.

Nitrogen fixation in endosymbiotic plant-bacterial associations is limited to the Fabid clade (e.g., squash, bean/pea and rose families). The most well-studied bacterial associations are between legumes and *Rhizobium.* The association between a nitrogen-fixing filamentous bacteria (*Frankia*) and a diverse range of trees and woody shrubs is less well characterized. However, a recent study found that a novel symbiotic factor from Frankia CcI3 strain was resistant to chitinase treatment and had relatively low molecular weight (i.e., in the range 0.5–5 KDa) (Chabaud et al., 2016). The novel symbiotic factor could trigger Ca^2+^ spikes in root hairs and induce *nodule inception (CgNIN)* gene expression in the actinorhizal plant *Casuarina glauca*. This finding suggests that certain symbiotic responses, such as Ca^2+^ spiking, are conserved across plants that can form symbiotic associations in the Fabid clade (Chabaud et al., 2016).

### Ca^2+^ channels and Ca^2+^ pumps involved in beneficial microbes triggered Ca^2+^ influxes

**Figure 1:**
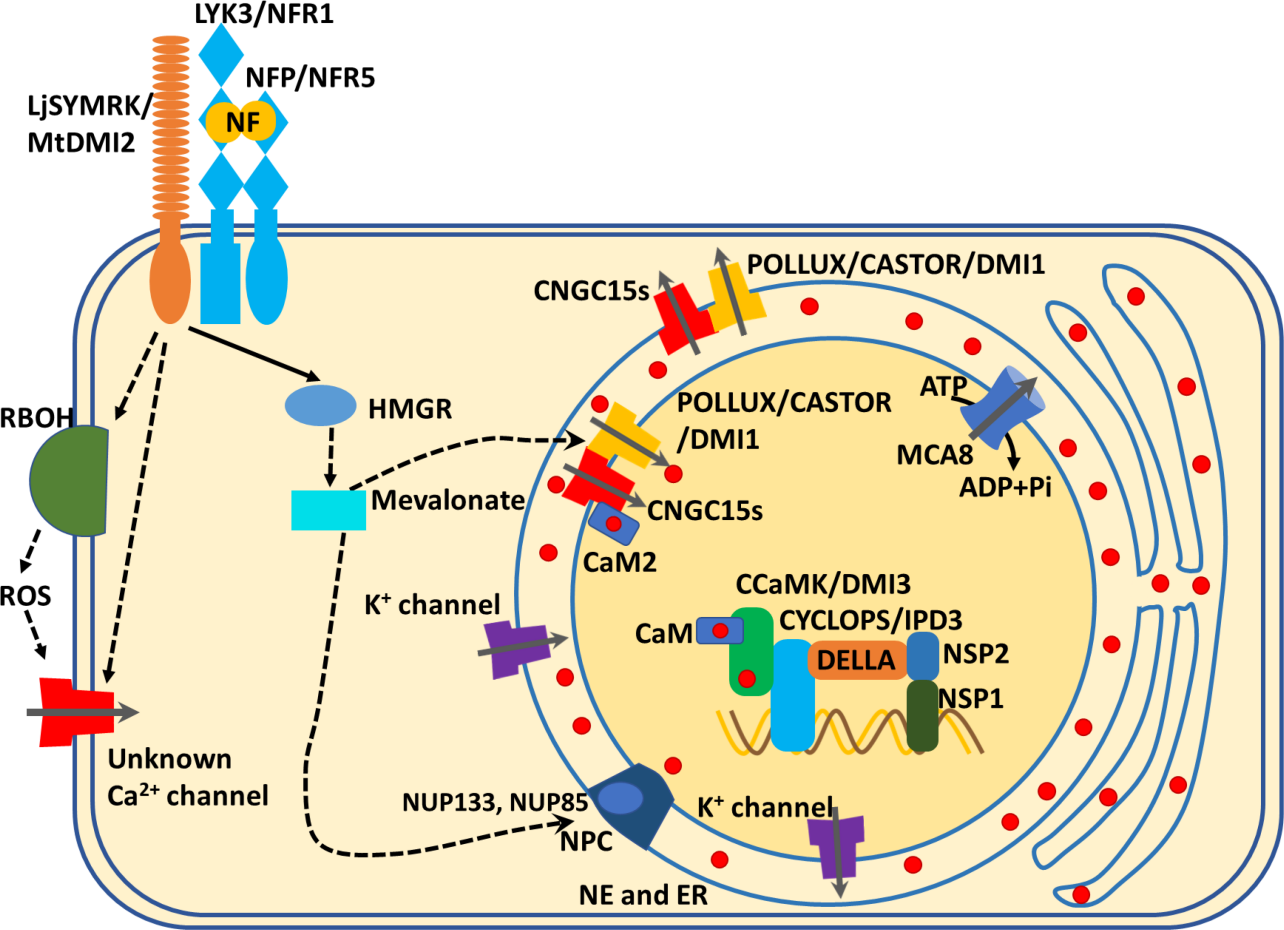
Ca^2+^ signals mediate local symbiotic signaling pathways in the root. Plants recognize the Nod factor *via* Nod factor perception (NFP)/Nod Factor Receptor 5 (NFR5) and LysM receptor kinase 3 (LYK3)/NFR1 subsequently activate the leucine-rich repeat receptor-like kinases which include the symbiotic receptor kinase *LjSYMRK* in *Lotus* and *MtDMI2* in *Medicago truncatula*. The activated *LjSYMRK*/*MtDMI2* may directly open unknown cytoplasmic membrane-localized Ca^2+^ channels or indirectly regulate Ca^2+^ channels through ROS signaling pathway, to induce cytosolic Ca^2+^ influxes. Meanwhile, *LjSYMRK*/*MtDMI2* interacts with 3-Hydroxy-3-Methylglutaryl Coenzyme A Reductase (HMGR) to initiate the biosynthesis of mevalonate. The mevalonate accumulation activates LjPOLLUX and LjCASTOR/MtDMI1. MtDM1/LjPOLLUX and LjCASTOR interact with the nuclear envelope (NE)-localized channels, CNGC15s (CNGC15a, b or c), to regulate Nod factor-induced Ca^2+^ inflex into the nucleus from the NE or endoplasmic reticulum. Ca^2+^-bound MtCaM2 interacts with CNGC15s, causing its closure and thus acting as a negative feedback loop for ion channels. Meanwhile, the nuclear localized Ca^2+^ pump, MtMCA8, uses ATP to transport the Ca^2+^ ions from the nucleus back to NE or ER to maintain the Ca^2+^ oscillation. In addition, a potential component of the nuclear pore complex (*NPC*), nucleoporins, such as *NUP133 and UNP85*, are essential for the Nod-factor-induced nuclear Ca^2+^ oscillation. The symbiotic Ca^2+^ signal is decoded by CaM to activate down-stream phosphorylation events, through the Ca^2+^- and CaM-dependent protein kinase, *LjCCaMK* or *MtDMI3*. The activated *LjCCaMK* or *MtDMI3* phosphorylates *LjCYCLOPS* or *MtIPD3*, which is a transcription factor. The phosphorylated *LjCYCLOPS* or *MtIPD3* associates with DELLA, *NSP2* and *NSP1* to form a complex, which binds to the promoter of symbiosis-associated genes to induce their expression, which ultimately leads to nodulation.

Since nuclear Ca^2+^ oscillations are required for rhizobial and mycorrhizal symbioses, studies to better understand Ca^2+^ oscillations have mainly focused on ion channels and a pump located at the nuclear envelope (NE). The Ca^2+^ channels include CNGCs among which CNGC15s is crucial for the observed Ca^2+^ spiking triggered by rhizobia colonization in *Medicago*. The CNGCs are regulated by calmodulin (CaM) *via* its interaction with the CNGC isoleucine glutamine (IQ) motif. A recent study found that Ca^2+^-bound CaM2 regulates Ca^2+^ spiking by associating with the CNGC15s (e.g., CNGC15a, CNGC15b, and CNGC15c), which results in closed Ca^2+^ channels. The closing of the channel prevents it from releasing Ca^2+^ into the nucleoplasm, while a calcium pump (MCA8) drives calcium back to the nuclear envelope lumen; the opening and closing of the channels shape the nucleoplasmic calcium concentration (Cerro et al., 2022). A mutated CaM2, called CaM2^R91A,^ displayed increased binding affinity to CNGC15s. When CaM2^R91A^ was expressed in *Medicago truncatula*, the plants exhibited an increased Ca^2+^ oscillation frequency during early stage of colonization in both AM and rhizobia. Moreover, plants expressing CaM2^R91A^ showed enhanced Nod-factor-mediated induction of nodulation-related genes, such as *NIN* and *NF YA1*. Although the CaM2^R91A^ expressing plants were able to maintain enhanced bacterial symbiosis at later timepoints (14 and 28 dpi), they could not sustain AM intraradical hyphae and arbuscule formation in the roots (Cerro et al., 2022). Thus, the Ca^2+^-bound form of CaM2 plays an important role in modulating CNGC15 activity and the subsequent Ca^2+^ oscillations, but the downstream Ca^2+^-mediated signaling networks differ between AM and root nodule symbiosis (**Figure 1**).

The nuclear pore complex (NPC) in *Lotus japonicus* is essential for Nod factor-induced nuclear Ca^2+^ oscillations (Kanamori et al., 2006). Nod factor triggered a weaker nuclear Ca^2+^ oscillation in mutant *nup133* as compared to the wild-type control, and the *nup133* mutants showed no mycorrhizal colonization and reduced nodulation by *Rhizobium* bacteria at permissive temperatures (Kanamori et al., 2006). In *L. japonicus*, the nucleoporin gene *NUP85* was also required for Nod-factor-induced nuclear Ca^2+^ oscillation as well as bacterial nodulation and mycorrhizal colonization (Saito et al., 2007). The nuclear pore complex may mediate Nod factor-induced nuclear Ca^2+^ oscillations indirectly by modulating the transport of symbiosis-related mRNAs [such as Nod receptors and symbiosis-related Ca^2+^ channels (CASTOR, POLLUX/DMI1, CNGC15) or Ca^2+^pumps (MCA8)] from nucleus to ribosome for polypeptides or protein biosynthesis. Another possibility is that the NPCs regulate the localization of CASTOR, POLLUX/DMI1, CNGC15 to the nuclear membranes, although the biological mechanism involved deserves further study.

In addition to nuclear-localized Ca^2+^ channels and components of the nuclear pore complex (NUP85, NUP133), other cation channels are also required for nuclear Ca^2+^ oscillations. *Medicago truncatula DMI1* and its two homologs CASTOR and POLLUX in *L. japonicus* were once thought to be potassium (K^+^) channels (Peiter et al., 2007; Charpentier et al., 2016) but more recent evidence shows that they were Ca^2+^ channels (Kim et al., 2019). Ca^2+^ binding to the CASTOR gating ring was required for root nodule symbiosis, and legumes carrying mutated CASTOR at either of two Ca^2+^ binding sites (D442A or E493Q) failed to form rhizobia-induced nodulation. This finding links defects in Ca^2+^ binding to Ca^2+^ channel regulation, which ultimately affects the legume-microbe symbiosis (Kim et al., 2019). However, this study was carried out in mammalian cells (HEK293), and to further characterize the function of *DMI1* and clarify if it is a Ca^2+^ or K^+^ channel, future research should be performed in plant cells. Furthermore, Nod factor induced the association between C-terminal of DMI1 and N-terminal of CNGC15s. In addition, DMI1 associated with CNGC15s (CNGC15a, CNGC15b, CNGC15c) to form a complex protein in nuclear membranes which was required for the activation of nuclear Ca^2+^ spiking (Charpentier et al., 2016). The latest study further confirmed that the two cation channels, DMI1 and CNGC15, form a channel complex to regulate nuclear symbiotic Ca^2+^ oscillations and nodule development (Liu et al., 2022). Genetic testing showed that gain-of-function mutations in *MtDMI1*, *DMI1* (S760N), displayed spontaneous nuclear Ca^2+^ spikes and constitutive activation of nodulation (Liu et al., 2022). The S760N mutation *DMI1* caused nuclear Ca^2+^ oscillations in a CNGC15 dependent manner and spontaneous nodulation (Liu et al., 2022). These studies extend our understanding of activating cation channels complex to form nuclear Ca^2+^ oscillations during plant symbiotic microbe interaction.

Ca^2+^ pumps regulate Ca^2+^ changes in the nuclear region, like nuclear localized Ca^2+^ channels, during plant and symbiotic microbe interactions. Other than depending on the ion concentration or electrochemical gradient like Ca^2+^ channels, Ca^2+^ pumps consumed ATP to facilitate Ca^2+^ movement against it (Demidchik et al., 2018). A Ca^2+^ pump, *MtMCA8*, was involved in the formation of symbiosis-induced nuclear Ca^2+^ oscillation, and *MCA8-*silenced plants displayed decreased mycorrhizal colonization (Capoen et al., 2011). Unlike *DMI1* being mainly distributed in the inner layer of nuclear membrane, *MCA8* was equally localized at both inner and outer layers of the nuclear membrane and at the endoplasmic reticulum (ER). A hypothesis proposes that the inner-layer-localized *MCA8* mediates the recapture of nuclear Ca^2+^ spikes, while the outer-layer- and the ER-localized *MCA8* may reload the Ca^2+^ store at the ER or nuclear envelope from the cytoplasm (Capoen et al., 2011; Tian et al., 2020).

A key component of calcium regulation during plant symbiosis is the small guanosine triphosphatase (GTPase). The GTPase, belongs to the Rho/Rop family, directly regulates reactive oxygen species (ROS) production through activating the respiratory burst oxidase homolog B (RBOHB) (Wang et al., 2020). The ROS activates Ca^2+^ channels and triggers Ca^2+^ influxes, which subsequently activates plant immune responses (Wang et al., 2020), which suggests the GTPases play a role in plant defense (Rivero et al., 2019). Interestingly, the small GTPases [Rho-like GTPas (*MtROPs*)] and heterotrimeric G-proteins including Gα, Gβ, and Gγ subunits are also involved in root nodule symbiosis (Ke et al., 2012; Pandey, 2019; Bovin et al., 2021): the expressions of *MtROP3*, *MtROP5* and *MtROP6* were induced in rhizobia-infected roots (Liu et al., 2010); and genetic tests indicated that the Gα repressed nodule development, while the Gβ, Gγ and RGS promoted nodule development (Choudhury and Pandey, 2013). Further study revealed that ROP6 interacted with NFR5, but not with NFR1, to positively regulate infection thread development and nodulation formation in soybean (Ke et al., 2012). Another study revealed that ROP9 interacted with RACK1 and regulated root nodule development (Gao et al., 2021). Active NFR1 phosphorylated the regulator of G-protein signaling (RGS) proteins, which deactivated Gα, a negative regulator of root nodulation (Choudhury and Pandey, 2015). More studies are needed to reveal how small GTPases, together with Nod factor receptor complex, regulate symbiotic cytoplasmic and/or nuclear Ca^2+^ spiking.

### Plants transduce and decode Ca^2+^ signals through CCaMK during symbiosis

In 1995, the Poovaiah laboratory cloned and characterized a novel protein kinase from lily, which turned out to be regulated by both Ca^2+^ and CaM. Hence, it was named Ca^2+^/CaM-dependent protein kinase (CCaMK; Patil et al., 1995). Unlike all the other Ca^2+^/CaM-dependent protein kinases (CaMKs) which were discovered in animal cells, the CCaMK reported from plants contained a C-terminal visinin-like domain, including three EF-hand motifs, which functioned as a Ca^2+^-sensitive molecular switch (Sathyanarayanan et al., 2001). The CCaMK involved in symbiosis is encoded by *DMI3* in *Medicago truncatula* and *LjCCaMK* in *L. japonicus* (Patil et al., 1995). CCaMK is essential for root nodule formation and mycorrhizal associations. CCaMK has a serine/threonine kinase domain at the N-terminal and two Ca^2+^-mediated regulatory domains at the C-terminal. The two C-terminal domains include a visinin-like domain with three EF-hand motifs (i.e., identified as three Ca^2+^-binding domains) and one CaM-binding domain with autoinhibitory function (Sathyanarayanan et al., 2001; Yuan et al., 2017). Plants carrying the mutated CCaMK lacking the autoinhibitory domain exhibited spontaneous nodulation even without rhizobia infection (Gleason et al., 2006). This data indicates that the legume CCaMK is a master controller of nodulation and its autoinhibitory domain is important in regulating its activity. Further studies have shown that when basal levels of Ca^2+^ bind to CCaMK, the protein is kept in an inactive state. However, at elevated Ca^2+^ concentrations (e.g., Ca^2+^ spiking), Ca^2+^/CaM also binds to CCaMK, and the protein becomes activated (Miller et al., 2013). Thus, CCaMK is kept in an inactive state when there are no symbiotic microbes present. However, once symbiosis signals are perceived, Ca^2+^ spiking is induced, and the inactive CCaMK state is overridden by higher levels of Ca^2+^ and CaM binding (Miller et al., 2013).

**Figure 2:**
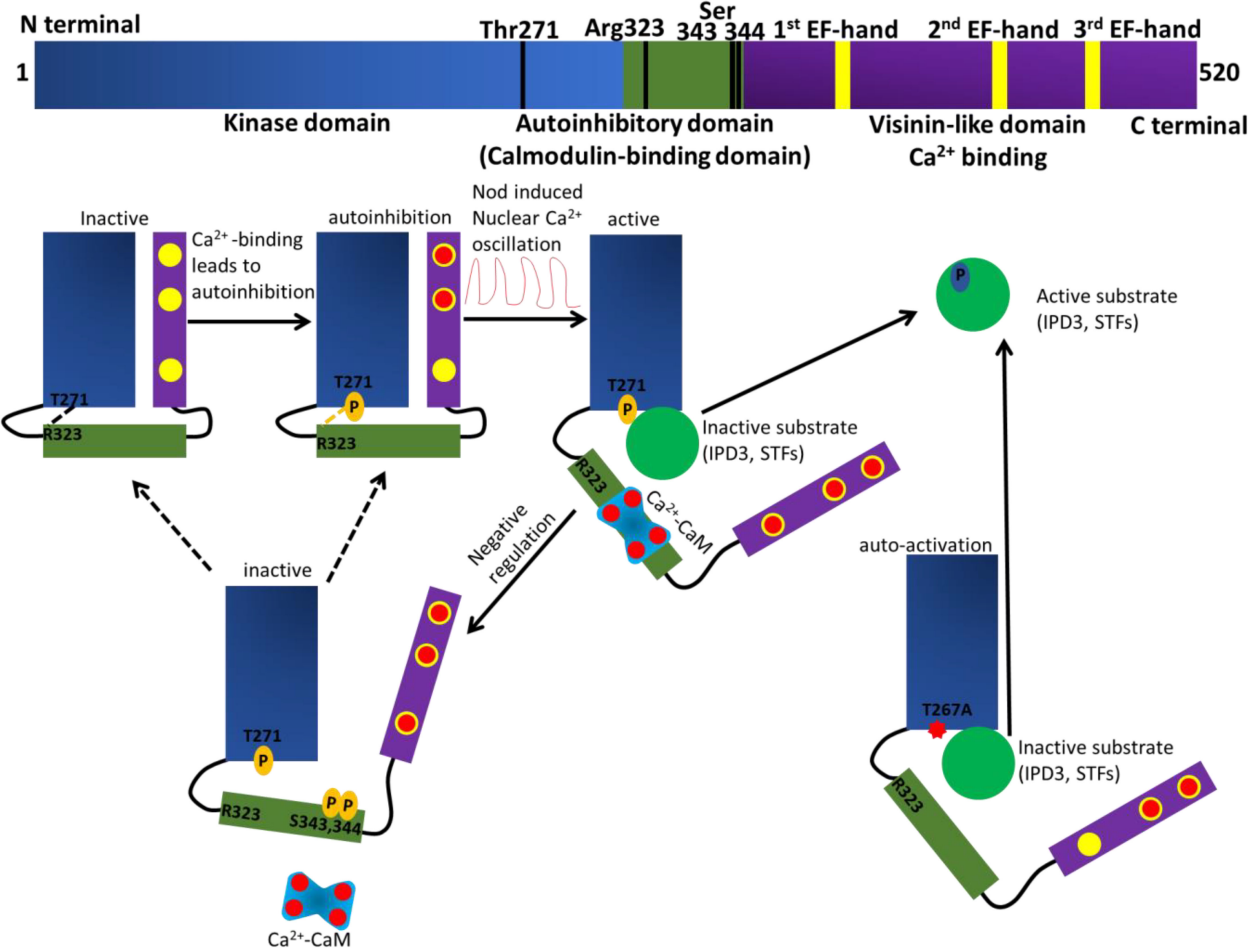
Schematic presentation displaying the domain structure of Ca^2+^/CaM-dependent protein kinases (CCaMK). The kinase domain (KD) is colored in blue, the autoinhibitory domain (AID) and calmodulin (CaM)-binding domain (CaMBD) are colored in green, the visinin-like domain (VID) is colored in purple and the EF-hand motifs in VID are in yellow. When symbiotic microbes are absent, the auto-inhibitory domain interacts with the kinase domain through a hydrogen bond between amino acid Thr-271 in KD and Arg-323 in AID. The interaction keeps the CCaMK inactive. The basal Ca^2+^ concentrations in root cell may bind to one or two EF-hands in the visinin-like domain, but not to all three EF-hand motifs. When symbiotic bacteria are present, the Nod factors induce nuclear Ca^2+^ oscillations. Hence, Ca^2+^ is loaded onto the EF-hand motifs in VID and to CaM. Subsequently, the Ca^2+^-loaded CaM will interact with the CCaMK AID. Ca^2+^ and CaM trigger conformational changes in the tertiary structure of CCaMK resulting in the AID being detached from the KD and removing the auto-inhibition caused by the Thr-271 phosphorylation. Thus, the CCaMK is completely activated. When the amino acid Ser-343 and/or Ser-344 in the AID are phosphorylated, the interaction between CCaMK and Ca^2+^-bound CaM is blocked and the CCaMK becomes inactive. The gain-of-function T271A mutant displays a spontaneous nodulation phenotype; a possible explanation is the hydrogen bond between amino acid Thr-271 in KD and Arg-323 in AID is compromised.

Further studies in the Poovaiah laboratory and others revealed that a mutated CCaMK negatively affects root nodule symbiosis in *Medicago truncatula* (Sinharoy et al., 2009; Jauregui et al., 2017). Site-directed mutations in the CaM-binding domain of CCaMK altered its binding capacity to CaM, providing an effective approach to study how CaM regulates CCaMK during rhizobial symbiosis in *Medicago truncatula*. Mutating the tryptophan at position 342 to phenylalanine (W342F) increased the CaM-binding capability of the mutant, which underwent autophosphorylation and catalyzed substrate phosphorylation in the absence of Ca^2+^ and CaM. When the mutant W342F was expressed in *ccamk-1* roots, the transgenic roots exhibited an altered nodulation phenotype. These results suggest that altering the CaM-binding domain of CCaMK could generate a constitutively activated kinase with a negative role in the physiological function of the CCaMK [(Jauregui et al., 2017) (**Figure 2**)].

The CCaMK phosphorylated symbiosis-related substrate has been identified, CYCLOPS in *Lotus* and interacting protein of DMI3 (IPD3) in *Medicago truncatula* (Lévy et al., 2004; Messinese et al., 2007; Yano et al., 2008). As with auto-active CCaMK, auto-active CYCLOPs causes spontaneous nodulation in the absence of rhizobia (Gleason et al., 2006; Hayashi et al., 2010; Miller et al., 2013; Singh et al., 2014). CCaMK phosphorylates CYCLOPS/IPD3 to form a complex that binds to promoter elements and induces the expression of symbiosis-involved genes (Yano et al., 2008; Singh et al., 2014). For example, in *Lotus*, CYCLOPS works with CCaMK and a DELLA transcription factor to regulate the expression of *reduced (or required) arbuscular mycorrhiza1* (*RAM1*) (Gobbato et al., 2012; Pimprikar et al., 2016). RAM1 is a GRAS transcription factor which, when expressed, initiates the colonization of plant roots by arbuscular mycorrhiza (Gobbato et al., 2012). During Nod-factor signaling, CCaMK/IPD3 forms large complexes with two GRAS proteins, nodulation signaling pathway1 (NSP1) and NSP2, in addition to DELLA proteins. The DELLA proteins work as scaffolding to link the CCaMK-IPD3 complex with the NSP1-NSP2 complex, resulting in a complicated unit that regulates symbiotic signaling (Fonouni-Farde et al., 2016; Jin et al., 2016). This unit also activates the expression of two downstream transcription factors, *NIN* and *ERN1* (ERF, *required for nodulation 1*) (Marsh et al., 2007). Furthermore, ERN1 and/or *ERN2* regulate the expression of *rhizobium-directed polar growth* (*RPG*), *cystathionine β-synthase like 1* (*CBS1*), *nodule pectate lyase* (*NPL*), and *nuclear factor YA 1 (NF-YA1)*, while *NIN* regulates the expression of *early nodulin 11 (ENOD11)* and *ENOD12* (Fonouni-Farde et al., 2016).

Another reported interactor of CCaMK is the Calf intestinal phosphatase 73 (*CIP73*) (Kang et al., 2011). CIP73 belongs to a large ubiquitin super family, and it contains a Scythe N ubiquitin-like domain. A report showed that CIP73 interacted with CCaMK in a Ca^2+^-independent manner (Kang et al., 2011). However, CIP73 is phosphorylated by CCaMK in a Ca^2+^/CaM-dependent manner (Kang et al., 2011). The *cip73* silencing mutants displayed significantly reduced nodulation as compared to the wild-type control, indicating that it has a role in nodule formation (Kang et al., 2011). Notably, due to CIP73 having a scythe-N ubiquitin-like domain, it may be interesting to study whether the 26S proteasome mediates rhizobial/AM fungal infections. Known components of the Ca^2+^-mediated local symbiotic pathway in roots is described in **Figures 1** and **2**.

### Phytohormone-mediates symbiosis through regulating the stability of the CCaMK-DELLA-CYCLOPS complex

To activate nodulation or arbuscule formation, the CaM-CCaMK-DELLA-CYCLOPS protein complex binds to the promoter of symbiosis-related genes (Pimprikar et al., 2016). The DELLAs are a key scaffold protein for symbiosis, but they are also critical transcription factors that regulate phytohormone signaling. Therefore, DELLAs may be the link between hormone signals and symbiosis (Liu et al., 2018). Studies about phytohormones involved in symbiosis focus on gibberellic acid (GA), auxin, cytokinin, and abscisic acid (ABA).

NFs-triggered the activation of nodulation requires an optimal level of GAs and exogenous high concentration (>0.01 μM) GA treatments inhibit AM and rhizobial symbioses (Ferguson et al., 2005; Ferguson et al., 2011). One hypothesis is that GA negatively regulates plant symbiosis through the disruption of the CCaMK-DELLA-CYCLOPS complex. This disruption occurs when GA-receptor GID1 (GA INSENSITIVE DWARF1) perceives GA, and then interacts with DELLA proteins (Nemoto et al., 2017). The GA-GID1-DELLA complex recruits a specific F-box protein that interacts with the SCF E3 ligase complex, resulting in the 26S proteasome-mediated ubiquitination and degradation of DELLA proteins. Recruiting E3 ligases to DELLAs (i.e., part of the CaM-CCaMK-DELLA-CYCLOPS complex) may lead to the degradation of the entire complex (Wang and Deng, 2011; Kudla et al., 2018).

Auxin has a positive role in nodulation (Suzaki et al., 2013; Breakspear et al., 2014; Bensmihen, 2015), and nodule numbers are regulated by shoot-to-root auxin transport (van Noorden et al., 2006). Auxin also seems to be positively involved in AM symbiosis (Hanlon and Coenen, 2011; Etemadi et al., 2014). However, a separate study revealed that indole-3-acetic acid (IAA), a class of auxin, promoted GA1 accumulation in pea (O’Neill and Ross, 2002); further studies are needed to extend our knowledge about GA and auxin crosstalk during plant symbiotic microbe interaction.

The role of Ca^2+^ in controlling cell division and growth is well recognized (Perris et al., 1968). It is becoming clear that there is also a linkage between cytokinin signaling and Ca^2+^ signaling. Cytokinin is an important hormone involved in symbiotic interactions between *Rhizobium* bacteria and leguminous plants. This interaction leads to the induction of the nitrogen-fixing nodule. It was proposed that cytokinin was the key differentiation signal for nodule organogenesis (Frugier et al., 2008). It was also proposed that cytokinin is involved in the regulation of *NIN* (Nodule Inception) expression to initiate nodule organogenesis and other transcriptional regulators through mechanisms operating both locally and systemically (Yano et al., 2008; Liu et al., 2019). Further study revealed that Ca^2+^ signaling involve cytokinin mediated nodule formation through regulating cytokinin biosynthesis (Reid et al., 2016; Reid et al., 2017). Nod factor induced cytokinin biosynthesis genes expression, including *isopentenyl transferase 2 (LjIPT2)* and *lonely guy 4 (LjLog4)*, and CCaMK is required for this induction (Reid et al., 2017), although the underlying mechanism is still unclear.

ABA application can inhibit root nodulation, suggesting that ABA is a negative regulator of rhizobial symbiosis (Suzuki et al., 2004; Ding et al., 2008). Interestingly, arbuscule formation was compromised in an ABA biosynthesis-defective tomato mutant *sitiens* (Herrera-Medina et al., 2007) and further work in *Medicago truncatula* supported the idea that some components of ABA signaling were needed for AM symbiosis (Charpentier et al., 2014). Another study revealed that ABA contributed to root symbiosis in a dose-dependent manner: high concentrations of ABA repressed AM colonization, while low ABA (i.e., less than 200 μM) promoted AM development (Liu et al., 2018). ABA works in complex signaling pathways with other hormones, including GA. In fact, the interconnection between ABA and GA is illustrated by ABA negatively regulating GA biosynthesis-related gene expression and positively regulating GA catabolism (Nag et al., 2005; Martín-Rodríguez et al., 2016). Another study revealed that exogenous ABA application enhanced the stability of DELLA protein, even in the presence of GA (Achard et al., 2006). ABA maintains the stability and integrity of DELLAs and low doses of ABA may contribute to its positive impact on AM symbiosis (Bedini et al., 2018). High levels of ABA impair Ca^2+^ oscillations, which negatively affects symbiosis (Charpentier et al., 2014). Further studies could address whether SA and JA are also involved in root symbiosis through stabilizing the DELLA protein, although the underlying molecular mechanism remains unclear (Liu et al., 2018).

CCaMK also has a positive role in ABA-mediated responses (Ni et al., 2019; Chen et al., 2021). Work in rice showed that the type C protein phosphate (PP2C), also known as PP45, negatively affected CCaMK activity by dephosphorylating T263. However, ABA induced H_2_O_2_ accumulation suppressed the transcriptional expression of PP45 (Ni et al., 2019). Although this work was performed in rice, which does not form symbiotic relationships with rhizobia, it would be interesting to hypothesize that ABA is involved in the mediation of root symbiosis through CCaMK. Further studies are needed to better understand the interaction between CCaMK and ABA and their role in symbiosis.

## Systemic symbiotic signaling

A number of studies have revealed that plants tightly regulate nodule development through a systemic signaling pathway (root-derived peptides and shoot-derived microRNA), also known as autoregulation of nodulation (AON) (Kassaw et al., 2015). During early rhizobial infection events, the small peptides, CLAVATA (CLV)/Embryo-surrounding region (CLE), accumulate in roots and are transported to the shoots through the xylem (Yamaguchi et al., 2016; Wang et al., 2022). The rhizobial-induced CLE (RIC) is recognized by a receptor complex in leaves, and this recognition initiates the biosynthesis of cytokinins and the shoot-derived microRNA, miR2111 (Kassaw et al., 2015; Gautrat et al., 2020; Okuma and Kawaguchi, 2021). The shoot-derived regulators are transported to the root through the phloem to repress or fine-tune nodule formation (Wang et al., 2022), although the role of Ca^2+^ signaling in these systemic regulators is not understood.

**Figure 3:**
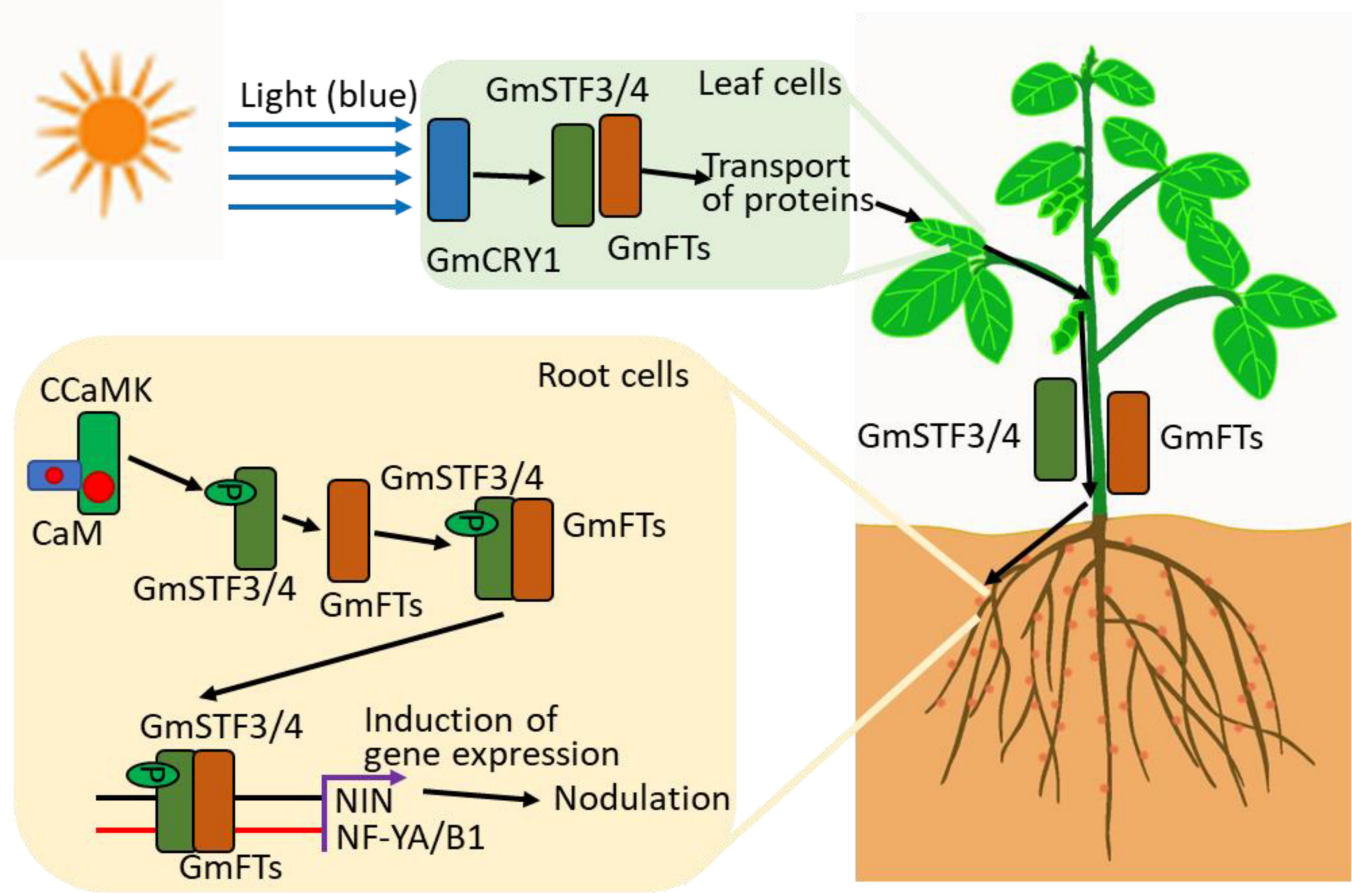
Systemic symbiotic signaling pathways in plants. Leaves perceive the blue light signal through the blue light receptor, *GmCRY*, which subsequently facilitates the long-distance transport of two symbiosis-associated transcription factors, *GmSTF3/4* and *GmFT*, from shoots to roots. CCaMK, a Ca^2+^ and CaM-binding protein phosphorylates *GmSTF3*, which then facilitates the interaction between *GmSTF3* and *GmFT* to form a complex. This complex binds to the promoter region of symbiosis-related genes, such as *NIN, NF-YA1* or *NF-YB1*, to trigger nodule formation in leguminous plant roots.

Recent studies indicate that photosynthesis and light signals participate in symbiotic nitrogen fixation in soybean through Ca^2+^ signaling. Root nodules formed when plants were grown under normal light conditions. However, when light was absent, nodule formation was disrupted. Moreover, root nodules were only formed when leaves were illuminated; only illuminating the roots failed to promote the formation of infection threads by rhizobia (Wang et al., 2021). Blue light was sufficient for nodule formation, and a known blue light receptor *GmGRY1* was required for light-induced nodulation. Light signals facilitated the movement and transportation of two proteins, *soybean TGACG-motif binding factor 3/4* (*GmSTF3/4*) and *flowering locus T* (*GmFTs*), from shoots to roots. Once these proteins are in roots, the transported *GmSTF3* is phosphorylated and becomes a substrate for the active CCaMK. The phosphorylated *GmSTF3* interacts with *GmFT2* to form a complex. This complex targets the promoter regions of *GmNF-YA1* and *GmNF-YB1* and induce their expression, ultimately resulting in nodule formation. Thus, these findings using soybeans suggest that plants could interpret light signals in leaves and then signal roots that photosynthesis-derived carbohydrates are available to support symbiosis and enhance nitrogen fixation in roots (**Figure 3**). It is worthwhile to test whether Ca^2+^ signaling mediates the activation and formation of mobile signals and to determine the long-distance signal transport.

## Summary and outlook

We are starting to learn the complexity of Ca^2+^ signaling during plant-microbe symbiotic interactions. Previous studies have mainly focused on the individual, local signaling components. A recent study Wang et al. (2021) uncovered that not only the local signaling, but also the systemic signal integration coordinately regulates symbiotic responses. Further studies suggest the specific spatial and temporal Ca^2+^ signaling response is tightly regulated and sophisticated; it is likely that multiple symbiotic signaling pathways are involved in fine-tuning a precise symbiosis response in plants (Kudla et al., 2018).

Although exciting advances in Ca^2+^-mediated symbiotic signaling pathways are rapidly expanding our knowledge about how plants mediate symbiotic interactions, some questions remain to be answered. One question is whether Nod factor or symbiotic microbes induce the cytosolic Ca^2+^ transients (although nuclear Ca^2+^ oscillation has been well documented) and which Ca^2+^ component(s), Ca^2+^ channel or Ca^2+^ pumps that are localized in the cytoplasmic membrane is/are involved in this biological process. Another question is whether CCaMK is involved in the transport of ammonia from roots to shoots. More questions remain as to whether other novel Ca^2+^ signaling proteins [e.g., CaM-like proteins (CML) or calcineurin B-like proteins (CBLs)-CBL-interacting protein kinases (CIPKs)] participate in symbiotic regulation. The answers to the above questions should provide new insights into nodulation and arbuscular mycorrhizal colonization. This knowledge would empower us to develop strategies to improve and manipulate plant-microbe symbioses and, thus, increase crop yield and agricultural productivity.

## Author contributions

PY, FL, CG and BP were involved in writing this review. All authors contributed to the article and approved the submitted version.

## Funding

USDA NIFA (Hatch Project 1015621 to BWP) and past support of the National Science Foundation (grants 1021344 and 1557813 to BWP) are acknowledged. This publication was partly supported by Provincial Natural Science Foundation of Hunan (2021JJ40241) and the National Technology System for Grape Industry (CARS-29-ZP-9). This publication was supported in part by the USDA National Institute of Food and Agriculture, Hatch project 1015621 to CG.

## Conflict of interest

The authors declare that the research was conducted in the absence of any commercial or financial relationships that could be construed as a potential conflict of interest.

## Publisher’s note

All claims expressed in this article are solely those of the authors and do not necessarily represent those of their affiliated organizations, or those of the publisher, the editors and the reviewers. Any product that may be evaluated in this article, or claim that may be made by its manufacturer, is not guaranteed or endorsed by the publisher.

## References

[B1] Achard P. Cheng H. De Grauwe L. Decat J. Schoutteten H. Moritz T.. (2006). Integration of plant responses to environmentally activated phytohormonal signals. Science 311 (5757), 91–94. doi: 10.1126/science.1118642 16400150

[B2] Ané J. M. Kiss G. B. Riely B. K. Penmetsa R. V. Oldroyd G. E. Ayax C.. (2004). Medicago truncatula DMI1 required for bacterial and fungal symbioses in legumes. Science 303 (5662), 1364–1367. doi: 10.1126/science.1092986 14963334

[B3] Aslam S. N. Newman M.-A. Erbs G. Morrissey K. L. Chinchilla D. Boller T.. (2008). Bacterial polysaccharides suppress induced innate immunity by calcium chelation. Curr. Biol. 18 (14), 1078–1083. doi: 10.1016/j.cub.2008.06.061 18639458

[B4] Bedini A. Mercy L. Schneider C. Franken P. Lucic-Mercy E. (2018). Unraveling the initial plant hormone signaling, metabolic mechanisms and plant defense triggering the endomycorrhizal symbiosis behavior. Front Plant Sci. 9, 1800. doi: 10.3389/fpls.2018.01800 30619390 PMC6304697

[B5] Bensmihen S. (2015). Hormonal control of lateral root and nodule development in legumes. Plants (Basel) 4 (3), 523–547. doi: 10.3390/plants4030523 27135340 PMC4844399

[B6] Berridge M. J. Bootman M. D. Roderick H. L. (2003). Calcium signalling: dynamics, homeostasis and remodelling. Nat. Rev. Mol. Cell Biol. 4 (7), 517–529. doi: 10.1038/nrm1155 12838335

[B7] Bi G. Su M. Li N. Liang Y. Dang S. Xu J.. (2021). The ZAR1 resistosome is a calcium-permeable channel triggering plant immune signaling. Cell. 184 (13), 3528–3541. doi: 10.1016/j.cell.2021.05.003 33984278

[B8] Bovin A. D. Pavlova O. A. Dolgikh A. V. Leppyanen I. V. Dolgikh E. A. (2021). The role of heterotrimeric G-protein beta subunits during nodulation in medicago truncatula gaertn and pisum sativum l. Front. Plant Sci. 12. doi: 10.3389/fpls.2021.808573 PMC8790031 35095980

[B9] Breakspear A. Liu C. Roy S. Stacey N. Rogers C. Trick M.. (2014). The root hair “Infectome” of medicago truncatula uncovers changes in cell cycle genes and reveals a requirement for auxin signaling in rhizobial infection. Plant Cell 26 (12), 4680–4701. doi: 10.1105/tpc.114.133496 25527707 PMC4311213

[B10] Capoen W. Sun J. Wysham D. Otegui M. S. Venkateshwaran M. Hirsch S.. (2011). Nuclear membranes control symbiotic calcium signaling of legumes. Proc. Natl. Acad. Sci. U. S. A. 108 (34), 14348–14353. doi: 10.1073/pnas.1107912108 21825141 PMC3161518

[B11] Cerro P.d. Cook N. M. Huisman R. Dangeville P. Grubb L. E. Marchal C.. (2022). Engineered CaM2 modulates nuclear calcium oscillation and enhances legume root nodule symbiosis. Proc. Natl. Acad. Sci. 119 (13), e2200099119. doi: 10.1073/pnas.2200099119 35324326 PMC9060481

[B12] Chabaud M. Gherbi H. Pirolles E. Vaissayre V. Fournier J. Moukouanga D.. (2016). Chitinase-resistant hydrophilic symbiotic factors secreted by frankia activate both Ca(2+) spiking and NIN gene expression in the actinorhizal plant casuarina glauca. New Phytol. 209 (1), 86–93. doi: 10.1111/nph.13732 26484850

[B13] Charpentier M. Sun J. Martins T. V. Radhakrishnan G. V. Findlay K. Soumpourou E.. (2016). Nuclear-localized cyclic nucleotide–gated channels mediate symbiotic calcium oscillations. Science 352 (6289), 1102–1105. doi: 10.1126/science.aae0109 27230377

[B14] Charpentier M. Sun J. Wen J. Mysore K. S. Oldroyd G. E. (2014). Abscisic acid promotion of arbuscular mycorrhizal colonization requires a component of the PROTEIN PHOSPHATASE 2A complex. Plant Physiol. 166 (4), 2077–2090. doi: 10.1104/pp.114.246371 25293963 PMC4256847

[B15] Chen M. Ni L. Chen J. Sun M. Qin C. Zhang G.. (2021). Rice calcium/calmodulin-dependent protein kinase directly phosphorylates a mitogen-activated protein kinase kinase to regulate abscisic acid responses. Plant Cell 33 (5), 1790–1812. doi: 10.1093/plcell/koab071 33630095 PMC8254507

[B16] Choudhury S. R. Pandey S. (2013). Specific subunits of heterotrimeric G proteins play important roles during nodulation in soybean. Plant Physiol. 162 (1), 522–533. doi: 10.1104/pp.113.215400 23569109 PMC3641229

[B17] Choudhury S. R. Pandey S. (2015). Phosphorylation-dependent regulation of G-protein cycle during nodule formation in soybean. Plant Cell 27 (11), 3260–3276. doi: 10.1105/tpc.15.00517 26498905 PMC4682299

[B18] Cui L. Guo F. Zhang J. Yang S. Meng J. Geng Y.. (2019). Synergy of arbuscular mycorrhizal symbiosis and exogenous Ca2+ benefits peanut (Arachis hypogaea l.) growth through the shared hormone and flavonoid pathway. Sci. Rep. 9 (1), 16281. doi: 10.1038/s41598-019-52630-7 31700111 PMC6838158

[B19] Demidchik V. Shabala S. Isayenkov S. Cuin T. A. Pottosin I. (2018). Calcium transport across plant membranes: Mechanisms and functions. New Phytol. 220 (1), 49–69. doi: 10.1111/nph.15266 29916203

[B20] Ding Y. Kalo P. Yendrek C. Sun J. Liang Y. Marsh J. F.. (2008). Abscisic acid coordinates nod factor and cytokinin signaling during the regulation of nodulation in medicago truncatula. Plant Cell 20 (10), 2681–2695. doi: 10.1105/tpc.108.061739 18931020 PMC2590744

[B21] Ehrhardt D. W. Wais R. Long S. R. (1996). Calcium spiking in plant root hairs responding to rhizobium nodulation signals. Cell 85 (5), 673–681. doi: 10.1016/S0092-8674(00)81234-9 8646776

[B22] Etemadi M. Gutjahr C. Couzigou J. M. Zouine M. Lauressergues D. Timmers A.. (2014). Auxin perception is required for arbuscule development in arbuscular mycorrhizal symbiosis. Plant Physiol. 166 (1), 281–292. doi: 10.1104/pp.114.246595 25096975 PMC4149713

[B23] Ferguson B. J. Foo E. Ross J. J. Reid J. B. (2011). Relationship between gibberellin, ethylene and nodulation in pisum sativum. New Phytol. 189 (3), 829–842. doi: 10.1111/j.1469-8137.2010.03542.x 21087260

[B24] Ferguson B. J. Ross J. J. Reid J. B. (2005). Nodulation phenotypes of gibberellin and brassinosteroid mutants of pea. Plant Physiol. 138 (4), 2396–2405. doi: 10.1104/pp.105.062414 16055684 PMC1183425

[B25] Fonouni-Farde C. Tan S. Baudin M. Brault M. Wen J. Mysore K. S.. (2016). DELLA-mediated gibberellin signalling regulates nod factor signalling and rhizobial infection. Nat. Commun. 7 (1), 12636. doi: 10.1038/ncomms12636 27586842 PMC5025792

[B26] Frugier F. Kosuta S. Murray J. D. Crespi M. Szczyglowski K. (2008). Cytokinin: Secret agent of symbiosis. Trends Plant Sci. 13 (3), 115–120. doi: 10.1016/j.tplants.2008.01.003 18296104

[B27] Gao J.-P. Xu P. Wang M. Zhang X. Yang J. Zhou Y.. (2021). Nod factor receptor complex phosphorylates GmGEF2 to stimulate ROP signaling during nodulation. Curr. Biol. 31 (16), 3538–3550.e3535. doi: 10.1016/j.cub.2021.06.011 34216556

[B28] Gautrat P. Laffont C. Frugier F. (2020). Compact root architecture 2 promotes root competence for nodulation through the miR2111 systemic effector. Curr. Biol. 30 (7), 1339–1345.e1333. doi: 10.1016/j.cub.2020.01.084 32109394

[B29] Gleason C. Chaudhuri S. Yang T. Munoz A. Poovaiah B. W. Oldroyd G. E. D. (2006). Nodulation independent of rhizobia induced by a calcium-activated kinase lacking autoinhibition. Nature 441 (7097), 1149–1152. doi: 10.1038/nature04812 16810256

[B30] Gobbato E. Marsh J. F. Vernié T. Wang E. Maillet F. Kim J.. (2012). A GRAS-type transcription factor with a specific function in mycorrhizal signaling. Curr. Biol. 22 (23), 2236–2241. doi: 10.1016/j.cub.2012.09.044 23122845

[B31] Granqvist E. Sun J. Op den Camp R. Pujić P. Hill L. Normand P.. (2015). Bacterial-induced calcium oscillations are common to nitrogen-fixing associations of nodulating legumes and non-legumes. New Phytol. 207 551–558. doi: 10.1111/nph.13464 26010117 PMC4736677

[B32] Haney C. H. Riely B. K. Tricoli D. M. Cook D. R. Ehrhardt D. W. Long S. R. (2011). Symbiotic rhizobia bacteria trigger a change in localization and dynamics of the medicago truncatula receptor kinase LYK3. Plant Cell 23 (7), 2774–2787. doi: 10.1105/tpc.111.086389 21742993 PMC3226205

[B33] Hanlon M. T. Coenen C. (2011). Genetic evidence for auxin involvement in arbuscular mycorrhiza initiation. New Phytol. 189 (3), 701–709. doi: 10.1111/j.1469-8137.2010.03567.x 21091696

[B34] Hassan S. Mathesius U. (2012). The role of flavonoids in root–rhizosphere signalling: Opportunities and challenges for improving plant–microbe interactions. J. Exp. Bot. 63 (9), 3429–3444. doi: 10.1093/jxb/err430 22213816

[B35] Hayashi T. Banba M. Shimoda Y. Kouchi H. Hayashi M. Imaizumi-Anraku H. (2010). A dominant function of CCaMK in intracellular accommodation of bacterial and fungal endosymbionts. Plant J. 63 (1), 141–154. doi: 10.1111/j.1365-313X.2010.04228.x 20409002 PMC2916219

[B36] Herrera-Medina M. J. Steinkellner S. Vierheilig H. Ocampo Bote J. A. García Garrido J. M. (2007). Abscisic acid determines arbuscule development and functionality in the tomato arbuscular mycorrhiza. New Phytol. 175 (3), 554–564. doi: 10.1111/j.1469-8137.2007.02107.x 17635230

[B37] Jauregui E. Du L. Gleason C. Poovaiah B. W. (2017). W342F mutation in CCaMK enhances its affinity to calmodulin but compromises its role in supporting root nodule symbiosis in medicago truncatula. Front. Plant Sci. 8. doi: 10.3389/fpls.2017.01921 PMC5696362 29201032

[B38] Jin Y. Liu H. Luo D. Yu N. Dong W. Wang C.. (2016). DELLA proteins are common components of symbiotic rhizobial and mycorrhizal signalling pathways. Nat. Commun. 7, 12433. doi: 10.1038/ncomms12433 27514472 PMC4990646

[B39] Kanamori N. Madsen L. H. Radutoiu S. Frantescu M. Quistgaard E. M. H. Miwa H.. (2006). A nucleoporin is required for induction of Ca^2+^ spiking in legume nodule development and essential for rhizobial and fungal symbiosis. Proc. Natl. Acad. Sci. 103 (2), 359–364. doi: 10.1073/pnas.0508883103 16407163 PMC1326171

[B40] Kang H. Zhu H. Chu X. Yang Z. Yuan S. Yu D.. (2011). A novel interaction between CCaMK and a protein containing the Scythe_N ubiquitin-like domain in lotus japonicus. Plant Physiol. 155 (3), 1312–1324. doi: 10.1104/pp.110.167965 21209278 PMC3046588

[B41] Kassaw T. Bridges W. Jr. Frugoli J. (2015). Multiple autoregulation of nodulation (AON) signals identified through split root analysis of medicago truncatula sunn and rdn1 mutants. Plants (Basel) 4 (2), 209–224. doi: 10.3390/plants4020209 27135324 PMC4844323

[B42] Ke D. Fang Q. Chen C. Zhu H. Chen T. Chang X.. (2012). The small GTPase ROP6 interacts with NFR5 and is involved in nodule formation in lotus japonicus. Plant Physiol. 159 (1), 131–143. doi: 10.1104/pp.112.197269 22434040 PMC3375957

[B43] Kevei Z. Lougnon G. Mergaert P. Horváth G. V. Kereszt A. Jayaraman D.. (2007). 3-hydroxy-3-methylglutaryl coenzyme a reductase 1 interacts with NORK and is crucial for nodulation in medicago truncatula. Plant Cell 19 (12), 3974–3989. doi: 10.1105/tpc.107.053975 18156218 PMC2217646

[B44] Kim S. Zeng W. Bernard S. Liao J. Venkateshwaran M. Ane J.-M.. (20193703). Ca2+-regulated Ca2+ channels with an RCK gating ring control plant symbiotic associations. Nat. Commun. 10 (1):1–12. doi: 10.1038/s41467-019-11698-5 PMC6697748 31420535

[B45] Köster P. DeFalco T. A. Zipfel C. (2022). Ca2+ signals in plant immunity. EMBO J. 41 (12), e110741. doi: 10.15252/embj.2022110741 35560235 PMC9194748

[B46] Kudla J. Becker D. Grill E. Hedrich R. Hippler M. Kummer U.. (2018). Advances and current challenges in calcium signaling. New Phytol. 218 (2), 414–431. doi: 10.1111/nph.14966 29332310

[B47] Laplaze L. Lucas M. Champion A. (2015). Rhizobial root hair infection requires auxin signaling. Trends Plant Sci. 20 (6), 332–334. doi: 10.1016/j.tplants.2015.04.004 25920666

[B48] Lefebvre B. Timmers T. Mbengue M. Moreau S. Hervé C. Tóth K.. (2010). A remorin protein interacts with symbiotic receptors and regulates bacterial infection. Proc. Natl. Acad. Sci. 107 (5), 2343–2348. doi: 10.1073/pnas.0913320107 20133878 PMC2836688

[B49] Lévy J. Bres C. Geurts R. Chalhoub B. Kulikova O. Duc G.. (2004). A putative Ca^2+^ and calmodulin-dependent protein kinase required for bacterial and fungal symbioses. Science 303 (5662), 1361–1364. doi: 10.1126/science.1093038 14963335

[B50] Liu W. Chen A. M. Luo L. Sun J. Cao L. P. Yu G. Q.. (2010). Characterization and expression analysis of medicago truncatula ROP GTPase family during the early stage of symbiosis. J. Integr. Plant Biol. 52 (7), 639–652. doi: 10.1111/j.1744-7909.2010.00944.x 20590994

[B51] Liu H. Lin J.-S. Luo Z. Sun J. Huang X. Yang Y.. (2022). Constitutive activation of a nuclear-localized calcium channel complex in *Medicago truncatula* . Proc. Natl. Acad. Sci. 119 (34), e2205920119. doi: 10.1073/pnas.2205920119 35972963 PMC9407390

[B52] Liu C. W. Murray J. D. (2016). The role of flavonoids in nodulation host-range specificity: An update. Plants (Basel) 5 (3). doi: 10.3390/plants5030033 PMC5039741 27529286

[B53] Liu J. Rutten L. Limpens E. van der Molen T. van Velzen R. Chen R.. (2019). A remote cis-regulatory region is required for NIN expression in the pericycle to initiate nodule primordium formation in medicago truncatula. Plant Cell 31 (1), 68–83. doi: 10.1105/tpc.18.00478 30610167 PMC6391699

[B54] Liu H. Zhang C. Yang J. Yu N. Wang E. (2018). Hormone modulation of legume-rhizobial symbiosis. J. Integr. Plant Biol. 60 (8), 632–648. doi: 10.1111/jipb.12653 29578639

[B55] Luan S. Wang C. (2021). Calcium signaling mechanisms across kingdoms. Annu. Rev. Cell Dev. Biol. 37, 311–340. doi: 10.1146/annurev-cellbio-120219-035210 34375534

[B56] Marsh J. F. Rakocevic A. Mitra R. M. Brocard L. Sun J. Eschstruth A.. (2007). Medicago truncatula NIN is essential for rhizobial-independent nodule organogenesis induced by autoactive calcium/calmodulin-dependent protein kinase. Plant Physiol. 144 (1), 324–335. doi: 10.1104/pp.106.093021 17369436 PMC1913781

[B57] Martín-Rodríguez J. A. Huertas R. Ho-Plágaro T. Ocampo J. A. Turečková V. Tarkowská D.. (2016). Gibberellin–abscisic acid balances during arbuscular mycorrhiza formation in tomato. Front. Plant Sci. 7. doi: 10.3389/fpls.2016.01273 PMC4993810 27602046

[B58] Ma Y. Zhao Y. Berkowitz G. A. (2017). Intracellular Ca2+ is important for flagellin-triggered defense in arabidopsis and involves inositol polyphosphate signaling. J. Exp. Bot. 68 (13), 3617–3628. doi: 10.1093/jxb/erx176 28595359 PMC5853439

[B59] Messinese E. Mun J. H. Yeun L. H. Jayaraman D. Rougé P. Barre A.. (2007). A novel nuclear protein interacts with the symbiotic DMI3 calcium- and calmodulin-dependent protein kinase of medicago truncatula. Mol. Plant Microbe Interact. 20 (8), 912–921. doi: 10.1094/mpmi-20-8-0912 17722695

[B60] Miller J. B. Pratap A. Miyahara A. Zhou L. Bornemann S. Morris R. J.. (2013). Calcium/Calmodulin-dependent protein kinase is negatively and positively regulated by calcium, providing a mechanism for decoding calcium responses during symbiosis signaling. Plant Cell 25 (12), 5053–5066. doi: 10.1105/tpc.113.116921 24368786 PMC3904005

[B61] Moscatiello R. Squartini A. Mariani P. Navazio L. (2010). Flavonoid-induced calcium signalling in rhizobium leguminosarum bv. viciae. New Phytol. 188 (3), 814–823. doi: 10.1111/j.1469-8137.2010.03411.x 20738787

[B62] Nag R. Maity M. K. DasGupta M. (2005). Dual DNA binding property of ABA insensitive 3 like factors targeted to promoters responsive to ABA and auxin. Plant Mol. Biol. 59 (5), 821–838. doi: 10.1007/s11103-005-1387-z 16270233

[B63] Nemoto K. Ramadan A. Arimura G. I. Imai K. Tomii K. Shinozaki K.. (2017). Tyrosine phosphorylation of the GARU E3 ubiquitin ligase promotes gibberellin signalling by preventing GID1 degradation. Nat. Commun. 8 (1), 1004. doi: 10.1038/s41467-017-01005-5 29042542 PMC5645313

[B64] Ni L. Fu X. Zhang H. Li X. Cai X. Zhang P.. (2019). Abscisic acid inhibits rice protein phosphatase PP45 *via* H(2)O(2) and relieves repression of the Ca(2+)/CaM-dependent protein kinase DMI3. Plant Cell 31 (1), 128–152. doi: 10.1105/tpc.18.00506 30538152 PMC6391686

[B65] Okuma N. Kawaguchi M. (2021). Systemic optimization of legume nodulation: A shoot-derived regulator, miR2111. Front. Plant Sci. 12. doi: 10.3389/fpls.2021.682486 PMC8321092 34335652

[B66] Oldroyd G. E. D. (2013). Speak, friend, and enter: Signalling systems that promote beneficial symbiotic associations in plants. Nat. Rev. Microbiol. 11 (4), 252–263. doi: 10.1038/nrmicro2990 23493145

[B67] O’Neill D. P. Ross J. J. (2002). Auxin regulation of the gibberellin pathway in pea. Plant Physiol. 130 (4), 1974–1982. doi: 10.1104/pp.010587 12481080 PMC166708

[B68] Panche A. N. Diwan A. D. Chandra S. R. (2016). Flavonoids: An overview. J. Nutr. Sci. 5, e47–e47. doi: 10.1017/jns.2016.41 28620474 PMC5465813

[B69] Pandey S. (2019). Heterotrimeric G-protein signaling in plants: Conserved and novel mechanisms. Annu. Rev. Plant Biol. 70, 213–238. doi: 10.1146/annurev-arplant-050718-100231 31035831

[B70] Patil S. Takezawa D. Poovaiah B. W. (1995). Chimeric plant calcium/calmodulin-dependent protein kinase gene with a neural visinin-like calcium-binding domain. Proc. Natl. Acad. Sci. U. S. A. 92 (11), 4897–4901. doi: 10.1073/pnas.92.11.4897 7761420 PMC41814

[B71] Peiter E. Sun J. Heckmann A. B. Venkateshwaran M. Riely B. K. Otegui M. S.. (2007). The medicago truncatula DMI1 protein modulates cytosolic calcium signaling. Plant Physiol. 145 (1), 192–203. doi: 10.1104/pp.107.097261 17631529 PMC1976572

[B72] Perris A. D. Whitfield J. F. TÖLg P. K. (1968). Role of calcium in the control of growth and cell division. Nature 219 (5153), 527–529. doi: 10.1038/219527a0 5668449

[B73] Pimprikar P. Carbonnel S. Paries M. Katzer K. Klingl V. Monica J.. (2016). A CCaMK-CYCLOPS-DELLA complex activates transcription of RAM1 to regulate arbuscule branching. Curr. Biol. 26 (8), 987–998. doi: 10.1016/j.cub.2016.01.069 27020747

[B74] Reid D. E. Heckmann A. B. Novák O. Kelly S. Stougaard J. (2016). CYTOKININ OXIDASE/DEHYDROGENASE3 maintains cytokinin homeostasis during root and nodule development in lotus japonicus. Plant Physiol. 170 (2), 1060–1074. doi: 10.1104/pp.15.00650 26644503 PMC4734552

[B75] Reid D. Nadzieja M. Novák O. Heckmann A. B. Sandal N. Stougaard J. (2017). Cytokinin biosynthesis promotes cortical cell responses during nodule development. Plant Physiol. 175 (1), 361–375. doi: 10.1104/pp.17.00832 28733389 PMC5580777

[B76] Rivero C. Traubenik S. Zanetti M. E. Blanco F. A. (2019). Small GTPases in plant biotic interactions. Small GTPases 10 (5), 350–360. doi: 10.1080/21541248.2017.1333557 28644721 PMC6748374

[B77] Saito K. Yoshikawa M. Yano K. Miwa H. Uchida H. Asamizu E.. (2007). NUCLEOPORIN85 is required for calcium spiking, fungal and bacterial symbioses, and seed production in lotus japonicus. Plant Cell 19 (2), 610–624. doi: 10.1105/tpc.106.046938 17307929 PMC1867344

[B78] Sathyanarayanan P. V. Siems W. F. Jones J. P. Poovaiah B. W. (2001). Calcium-stimulated autophosphorylation site of plant chimeric calcium/calmodulin-dependent protein kinase. J. Biol. Chem. 276 (35), 32940–32947. doi: 10.1074/jbc.M009648200 11399751

[B79] Sharma V. Bhattacharyya S. Kumar R. Kumar A. Ibañez F. Wang J.. (2020). Molecular basis of root nodule symbiosis between bradyrhizobium and ‘Crack-entry’ legume groundnut (Arachis hypogaea l.). Plants 9 (2), 276. doi: 10.3390/plants9020276 PMC7076665 32093403

[B80] Singh S. Katzer K. Lambert J. Cerri M. Parniske M. (2014). CYCLOPS , A DNA-Binding Transcriptional Activator, Orchestrates symbiotic root nodule development. Cell Host Microbe 15 (2), 139–152. doi: 10.1016/j.chom.2014.01.011 24528861

[B81] Sinharoy S. Saha S. Chaudhury S. R. DasGupta M. (2009). Transformed hairy roots of arachis hypogea: A tool for studying root nodule symbiosis in a non–infection thread legume of the aeschynomeneae tribe. Mol. Plant-Microbe Interact® 22 (2), 132–142. doi: 10.1094/mpmi-22-2-0132 19132866

[B82] Suzaki T. Ito M. Kawaguchi M. (2013). Induction of localized auxin response during spontaneous nodule development in lotus japonicus. Plant Signal Behav. 8 (3), e23359. doi: 10.4161/psb.23359 23299335 PMC3676504

[B83] Suzuki A. Akune M. Kogiso M. Imagama Y. Osuki K. Uchiumi T.. (2004). Control of nodule number by the phytohormone abscisic acid in the roots of two leguminous species. Plant Cell Physiol. 45 (7), 914–922. doi: 10.1093/pcp/pch107 15295075

[B84] Tian W. Wang C. Gao Q. Li L. Luan S. (2020). Calcium spikes, waves and oscillations in plant development and biotic interactions. Nat. Plants 6 (7), 750–759. doi: 10.1038/s41477-020-0667-6 32601423

[B85] van Noorden G. E. Ross J. J. Reid J. B. Rolfe B. G. Mathesius U. (2006). Defective long-distance auxin transport regulation in the medicago truncatula super numeric nodules mutant. Plant Physiol. 140 (4), 1494–1506. doi: 10.1104/pp.105.075879 16489131 PMC1435797

[B86] Venkateshwaran M. Jayaraman D. Chabaud M. Genre A. Balloon A. J. Maeda J.. (2015). A role for the mevalonate pathway in early plant symbiotic signaling. Proc. Natl. Acad. Sci. U. S. A. 112 (31), 9781–9786. doi: 10.1073/pnas.1413762112 26199419 PMC4534228

[B87] Wang F. Deng X. W. (2011). Plant ubiquitin-proteasome pathway and its role in gibberellin signaling. Cell Res. 21 (9), 1286–1294. doi: 10.1038/cr.2011.118 21788985 PMC3193469

[B88] Wang D. Dong W. Murray J. Wang E. (2022). Innovation and appropriation in mycorrhizal and rhizobial symbioses. Plant Cell 34 (5), 1573–1599. doi: 10.1093/plcell/koac039 35157080 PMC9048890

[B89] Wang T. Guo J. Peng Y. Lyu X. Liu B. Sun S.. (2021). Light-induced mobile factors from shoots regulate rhizobium-triggered soybean root nodulation. Science 374 (6563), 65–71. doi: 10.1126/science.abh2890 34591638

[B90] Wang R. He F. Ning Y. Wang G.-L. (2020). Fine-tuning of RBOH-mediated ROS signaling in plant immunity. Trends Plant Sci. 25 (11), 1060–1062. doi: 10.1016/j.tplants.2020.08.001 32861572

[B91] Yamaguchi Y. L. Ishida T. Sawa S. (2016). CLE peptides and their signaling pathways in plant development. J. Exp. Bot. 67 (16), 4813–4826. doi: 10.1093/jxb/erw208 27229733

[B92] Yano K. Yoshida S. Müller J. Singh S. Banba M. Vickers K.. (2008). CYCLOPS, a mediator of symbiotic intracellular accommodation. Proc. Natl. Acad. Sci. 105 (51), 20540–20545. doi: 10.1073/pnas.0806858105 19074278 PMC2629324

[B93] Yuan P. Du L. Poovaiah B. (2018a). Ca2+/Calmodulin-dependent AtSR1/CAMTA3 plays critical roles in balancing plant growth and immunity. Int. J. Mol. Sci. 19 (6):1–18. doi: 10.3390/ijms19061764 PMC6032152 29899210

[B94] Yuan P. Jauregui E. Du L. Tanaka K. Poovaiah B. W. (2017). Calcium signatures and signaling events orchestrate plant–microbe interactions. Curr. Opin. Plant Biol. 38, 173–183. doi: 10.1016/j.pbi.2017.06.003 28692858

[B95] Yuan P. Jewell J. B. Behera S. Tanaka K. Poovaiah B. W. (2020). Distinct molecular pattern-induced calcium signatures lead to different downstream transcriptional regulations *via* AtSR1/CAMTA3. Int. J. Mol. Sci. 21 (21), 1–17. doi: 10.3390/ijms21218163 PMC7662696 33142885

[B96] Yuan P. Poovaiah B. W. (2022). Interplay between Ca2+/Calmodulin-mediated signaling and AtSR1/CAMTA3 during increased temperature resulting in compromised immune response in plants. Int. J. Mol. Sci. 23 (4):1–17. doi: 10.3390/ijms23042175 PMC8880272 35216293

[B97] Yuan P. Tanaka K. Poovaiah B. W. (2021). Calmodulin-binding transcription activator AtSR1/CAMTA3 fine-tunes plant immune response by transcriptional regulation of the salicylate receptor NPR1. Plant Cell Environ. 44 (9), 3140–3154. doi: 10.1111/pce.14123 34096631

[B98] Yuan P. Yang T. Poovaiah B. W. (2018b). Calcium signaling-mediated plant response to cold stress. Int. J. Mol. Sci. 19 (12):1–11. doi: 10.3390/ijms19123896 PMC6320992 30563125

